# The Prevalence of Sexsomnia in a General Population Sample

**DOI:** 10.1007/s10508-025-03235-x

**Published:** 2025-10-03

**Authors:** Ståle Pallesen, Ingvild West Saxvig, Siri Waage, Carlos H. Schenck, Bjørn Bjorvatn

**Affiliations:** 1 Department of Psychosocial Science, Faculty of Psychology, University of Bergen, Christiesgt. 12, 5015 Bergen, Norway; 2 Norwegian Competence Center for Sleep Disorders, Haukeland University Hospital, Bergen, Norway; 3 Minnesota Regional Sleep Disorders Centre and Department of Psychiatry, Hennepin County Medical Center, Minneapolis, MN USA; 4 Medical School, University of Minnesota, Minneapolis, MN USA; 5 Department of Global Public Health and Primary Care, University of Bergen, Bergen, Norway

**Keywords:** Fondling, Intercourse, Masturbation, Parasomnia, Sexsomnia, Sexual behaviors

## Abstract

Few studies have surveyed the prevalence of sexsomnia and sexsomnic behaviors in general population samples. In the present study, 1002 respondents (508 males and 494 females), mean age 50.3 years (SD = 17.5), recruited from a Norwegian survey panel, participated in an online survey about sleep phenomena and sleep habits. The survey included questions about lifetime and current parasomnias, such as sleepwalking, sleep terrors, confusional arousals, and dream enactment, as well as sleep duration and sleep need. Questions about lifetime and current sexsomnia, various sexsomnic behaviors, and frequency of current sexsomnia episodes were included. Logistic regression analyses were conducted to identify predictors of lifetime and current sexsomnia. A total of 10.5% and 6.1% reported lifetime and current sexsomnia, respectively. In the adjusted analysis, male sex (OR = 1.58; 95% CI = 1.03–2.42), sleep terrors (OR = 2.80; 95% CI = 1.64–4.78), and dream enactment (OR = 2.46; 95% CI = 1.56–3.88) were significant predictors for lifetime sexsomnia, whereas sleep terrors (OR = 2.86; 95% CI = 1.48–5.51), and dream enactment (OR = 1.97; 95% CI = 1.10–3.51) were significant predictors for current sexsomnia. Among those who reported lifetime sexsomnia, 6.5% had current sexsomnia episodes with at least a weekly frequency. Masturbation (5.4%), and fondling (4.0%) were the two most common behaviors, whereas consummated intercourse (1.8%) was the least common sexsomnic behavior. Most participants with sexsomnia reported a relatively restricted range of sexsomnic behaviors. The results are discussed in light of the existing literature. Recommendations for future research are provided.

## Introduction

Parasomnias are one of six main categories of sleep disorders and are characterized by undesirable physical events or experiences occurring during entry into sleep, within sleep, or during arousal from sleep. This main sleep disorder category is further subdivided into non-rapid eye movement parasomnias (NREM-related parasomnias), rapid eye movement parasomnias (REM-related parasomnias), and other parasomnias, the latter not arising from any particular sleep stage (American Academy of Sleep Medicine, 2023). The former subtype represents incomplete arousals from slow wave sleep (SWS), also termed stage 3 (N3) sleep, and increased frequency of arousals from SWS seems to be a hallmark of NREM-related parasomnias (Espa et al., 2000). The diagnostic criteria for NREM-related parasomnia encompass episodes of incomplete awakenings from sleep, inappropriate or absent responsiveness to others, limited/no associated cognition or dream imagery, and partial/complete amnesia for the episode (American Academy of Sleep Medicine, 2023).

Confusional arousals comprise a type of NREM-related parasomnia characterized by mental confusion or confused behavior occurring in bed and an absence of terror or ambulation outside of the bed. One official subtype of confusional arousals is named “sleep related abnormal sexual behaviors”, also called atypical sexual behavior during sleep, sexsomnia or sleep sex. Behavioral manifestations of sexsomnia range from masturbation, fondling, initiation of intercourse, consummated intercourse, spontaneous orgasms to sexual vocalizations (American Academy of Sleep Medicine, 2023). Sometimes the behavior during sexsomnia deviates from wake sexual behavior, where some become more gentle and passionate, whereas others act more aggressively and even violently towards their partner (Andersen et al., 2024). Sexsomnia is also recognized as a diagnostic entity in the fifth and current edition of the *Diagnostic and Statistical Manual of Mental Disorders* (DSM-5). According to the DSM-5, NREM movement sleep arousal disorders are listed with sleepwalking as a subtype, and with a further option to specify if it occurs with sleep-related sexual behavior/sexsomnia (American Psychiatric Association, 2013). However, the 11th edition of the *International Classification of Diseases* does not include sleep-related sexual behavior/sexsomnia (World Health Organization, 2019/2021). In addition to the aforementioned diagnostic criteria, sexsomnia may also occur in cases of parasomnia overlap disorder, consisting of REM sleep behavior disorder (RBD) and a concurrent NREM-related parasomnia (Soca et al., 2016).

Among those affected by sexsomnia, various triggers/situational factors seem to be in play. These entail time from sleep onset, as most NREM-related parasomnias typically occur 0.5–1 h after sleep onset, and rarely later than 2–3 h after sleep onset (Rosenfeld & Elhajjar, 1998). Another trigger is sleep loss, which results in increase of the sleep homeostatic pressure (Pilon et al., 2008). Stress has, to an increasing degree, received attention as a trigger of NREM-related parasomnias, which most likely make the SWS unstable (Bargiotas et al., 2017). The presence of other sleep disorders that may cause awakenings from SWS, such as obstructive sleep apnea (OSA), restless legs syndrome, and periodic leg movement during sleep may also act as triggers (Andersen et al., 2024). However, one paper showed that OSA was not associated with increased risk of sexsomnia (Lundetræ et al., 2018). Nevertheless, well-documented cases of OSA triggering sexsomnia have been reported, with resolution of both conditions with control of the OSA (Khawaja et al., 2017; Schenck, 2015; Soca et al., 2016).

Some argue, based on reviews of the literature, that alcohol is a not a trigger of parasomnias (Pressman et al., 2007; Rumbold et al., 2014), and the American Academy of Sleep Medicine (2023) states that a disorder of arousal cannot be diagnosed in the context of alcohol intoxication. Still, it is likely, based on more recent reports, that modest use of alcohol for some people acts as a trigger for NREM-related parasomnias (Maschauer et al., 2017). Furthermore, certain medications, including sodium oxybate (Devesa et al., 2016) and zolpidem (Braga et al., 2024), have been linked with the onset of sexsomnia.

For those where sexsomnia involves another person, the mere presence of such a person in the immediate surroundings (e.g., same bed) seems to be a triggering factor (Andersen et al., 2024). Regarding background variables associated with sexsomnia, several studies attest to male preponderance (Andersen et al., 2024; Cankardas & Schenck, 2021; Chung et al., 2010). Studies have also shown that sexsomnia seems to share several sleep abnormalities with other NREM-related parasomnias, e.g., sleepwalking and sleep terrors, such as higher N3 fragmentation index, slow/mixed N3 arousal index, and elevated number of eye openings during N3 interruptions (Rossi et al., 2023). Beyond these factors, some patients report drug use and sexual dreams as precipitating factors (Cankardas & Schenck, 2021; Trajanovic et al., 2007).

Both in clinical or legal settings it is important, although sometimes difficult, to differentiate between sexsomnia and more common behaviors such as accidental touching the bed partner as well as so-called “wet dreams.” According to a small study of male students, the latter can result in ejaculations in more than 50% (Yu & Fu, 2011). Cross-cultural accounts further attest to the frequency of wet dreams and nocturnal emissions (Janssen, 2007). Another differential diagnosis is RBD, which may also entail sleep related sexual behavior, although such behavior is very rare in RBD (Muza et al., 2016). In a few cases, sleep related sexual behavior may reflect epileptic activity (Voges et al., 2019). These cases seem mainly to involve masturbation and ictal orgasms, and not completion of sexual intercourse. Further, these events tend also to occur out of wakefulness, are typically associated with nonsexual manifestations of epilepsy, and the behavior pattern is often stereotypical (Schenck, 2015; Voges et al., 2019).

Sexsomnia may for some people lead to legal consequences, and several recommendations have been published regarding how alleged sexsomnia may be evaluated, typically emphasizing constitutional as well as the critical situational factors (Bonkalo, 1974; Cramer Bornemann & Schenck, 2025; Cramer Bornemann et al., 2006; Fenwick, 1996; Mahowald et al., 1990; Morrison et al., 2014). Still, international multidisciplinary consensus on forensic evaluation of sexsomnia is lacking (Andersen et al., 2024). It is assumed that sexsomnia is underreported due to shame and stigma, and lack of tracking by both patients and practitioners, as well as amnesia that characterizes the episodes (Andersen et al., 2024). A recent review of the world literature on sexsomnia found 220 clinical cases from 15 countries and 5 continents, with 84% male predominance, an age range of 14–77 years, frequent complex NREM parasomnia histories, and 8 cases of sexual behaviors documented during video-polysomnography (Cramer Bornemann & Schenck, 2025).

A few studies on the prevalence of sexsomnia have been conducted. In a previous representative study of 1000 Norwegians, 18–96 years old, asking whether they had performed sexual acts in their sleep, 7.1% reported lifetime occurrence, 2.7% at least once during the last three months, and 0.4% current and ongoing at least once a week (Bjorvatn et al., 2010). In a study of 4372 patients referred for respiratory polysomnography due to suspicion of OSA, 3.1% reported having performed sexual acts during sleep during the last three months (Lundetræ et al., 2018). Among 823 sleep clinic patients, sexsomnia, defined as having initiated sexual acts (such as masturbating or initiating intercourse with a bed partner), was reported by 11.0% of the male and 4.0% of the female patients (Chung et al., 2010). Based on a convenience sample of 279 volunteers in Turkey, 15.3% reported sexsomnia. Of those with sexsomnia, more than 30% of the females reported masturbation, intercourse and sexual movements, whereas more than 40% of the males reported fondling, intercourse and sexual movements (Cankardas & Schenck, 2021). In an internet survey of 409 people who had visited a website for sexsomnia, 219 provided valid answers. Among the women, 68.7% reported masturbation, 53.7% fondling, 35.8% intercourse, 41.8% sexualized vocalization, and 35.8% sexualized movements. For males, the corresponding proportions were 34.2, 80.3, 59.2, 33.6, and 60.5%. For 3.0% of the females and 8.6% of the males, sexsomnia led to legal repercussions (Trajanovic et al., 2007). Among 41 sleep disorders center patients (37 men) with sexsomnia, Muza et al. (2016) found that 50.0% reported intercourse, 25.0% reported fondling and 17.9% reported masturbation in sleep, whereas 3.6% reported pelvis thrusting and sexual talking, respectively. Most previous studies have typically addressed specialized (e.g., clinical) samples. Further, samples sizes have throughout been small. Against this backdrop, we conducted a study in a large general population sample aiming to assess the prevalence and frequency of various sexsomnic behaviors. The aim of the study was also to identify relevant predictors of sexsomnia. We expected to find a higher prevalence of sexsomnia among males than females, and that sexsomnia would be associated with other NREM-related parasomnias.

## Method

### Participants

The unweighted sample comprised 508 (50.7%) males and 494 females (49.3%). The mean age was 50.3 years (SD = 17.5). When weights were applied, the sex distribution changed to 50.1% males and 49.9% females, and the mean age to 48.8 (SD = 17.9) years.

### Procedure

The data collection was conducted by Opinion, which is a leading polling agency in Norway. Potential respondents were recruited from a panel of participants, who had provided consent to take part in market research and various polls. Data were collected during September 2024. A total of 5055 panel members were invited to partake in an online survey about various sleep phenomena and sleep habits in the Norwegian population. A total of 1002 completed the survey, amounting to a response rate of 19.8%. Weights were calculated to adjust for discrepancies among age, sex, and geography of the sample, and age, sex, and geography of the adult population in Norway 18 years and above. The weights applied ranged from 0.51 to 2.05.

### Measures

#### Demographics

Questions were asked about age and sex.

#### Parasomnias

Questions about various parasomnias were asked: “Have you ever experienced or been told that you: 1) Have been sleepwalking, 2) Have woken up at night in a confusional state without remembering the event the next day (confusional arousal), 3) Have woken up at night in a terror without remembering the event the next day (sleep terrors), and 4) Have been acting out of a dream in your sleep (dream enactment). Response alternatives were “yes”/”no”. Those who answered “yes” were asked to indicate how often it had occurred during the last three months (“Never”, “Less than once per month”, “Less than once per week”, “1–2 days per week”, “3–5 days per week”, and “Daily or almost daily”).

#### Sexsomnia

One overall question was asked: “Have you ever experienced or been told that you have performed sexual acts in your sleep” (“yes/no/will not answer”). If endorsed, the respondent was asked how often it had occurred during the last three months (“Never”, “Less than once per month”, “Less than once per week”, “1–2 days per week”, “3–5 days per week”, and “Daily or almost daily”). In addition, six questions about specific sexsomnic behaviors were asked: Have you ever: 1) Masturbated in sleep (beyond accidentally touching your genitals)? 2) Fondled others (beyond accidentally touching them) in your sleep? 3) Tried to initiate intercourse with others in your sleep? 4) Initiated and consummated intercourse with others in your sleep? 5) Talked sexually in your sleep? 6) Performed sexualized acts (such as pelvis thrusts) in your sleep? The response alternatives were “No”, “Yes, on average less than once per year”, “Yes, on average 1–11 times per year”, “Yes, on average 1–3 times per month”, “Yes, on average one day per week or more often”, and “Unsure/don’t want to respond”.

#### Sleep Debt

The participants were asked about sleep duration on weekdays and sleep duration on weekends as well as a general question about sleep need. A sleep debt variable was then calculated by subtracting the sleep duration ((TST weekdays × 5 + TST weekends × 2)/7) from the sleep need representing the mean daily sleep deficit. This variable was recoded into “no sleep debt”, “modest sleep debt” (0.1–59.9 min), and “large sleep debt” (60 min or more).

### Statistical Analysis

The analyses were performed by IBM SPSS, version 29.0. Descriptive statistics in terms of means and standard deviations (interval and ratio variables), and percentages and 95% confidence interval (for sexsomnia prevalences), were reported. Logistic regression analyses for lifetime sexsomnia (no = 0, yes = 1) and current sexsomnia (no = 0, yes = 1) were performed, where age, sex (female = 0, male = 1), lifetime presence of sleep walking, sleep terrors, confusional arousal, dream enactment (no = 0, yes = 1), and sleep debt (no debt comprised the contrast category) comprised the independent variables. As all variance inflation factors were below 2.0, there was no violation of the non-multicollinearity assumption. Further, the frequency of sexsomnia the last three months among the participants with lifetime sexsomnia, was enumerated. Lifetime prevalence of the six sexsomnia subtypes were compared for men and women with sexsomnia, and their frequencies were also calculated for the sample as a whole. The proportion with zero to all six of the specific sexsomnias was also calculated. All analyses were conducted using weights.

## Results

Table 1 presents the means/standard deviations or percentages for the main study variables. Lifetime prevalence for sexsomnia was 10.5% (95% CI = 8.6–12.4%), whereas the prevalence for current (last 3 months) was 6.1% (95% CI = 4.6%−7.6%). Table 2 shows the results for the logistic regression analysis for lifetime sexsomnia. In the crude analysis, male sex (OR = 1.73), sleep walking (OR = 1.79), sleep terrors (OR = 3.87), confusional arousals (OR = 2.12), and dream enactment (OR = 3.13) were significant independent predictors. In the adjusted model, which overall was significant (χ^2^ = 53.92, df = 8, *p* < 0.01, Nagelkerke R^2^ = 0.11), male sex (OR = 1.58), sleep terrors (OR = 2.80), and dream enactment (OR = 2.46) remained significant. Table 3 presents the results for the logistic regression analysis for current (last three months) sexsomnia. Three independent variables, sleep terrors (OR = 3.74), confusional arousal (OR = 2.09), and dream enactment (OR = 2.55) were significant. In the adjusted model, which overall was significant (χ^2^ = 24.96, df = 8, *p* < 0.01, Nagelkerke R^2^ = 0.07), sleep terrors (OR = 2.86), and dream enactment (OR = 1.97) remained significant. In terms of frequency of current sexsomnia, the results shown in Table 4 demonstrate that about 42% of participants with lifetime sexsomnia had not experienced any episode during the last 3 months, 42% had experienced episodes during the last three months with an occurrence of less than monthly, and 6.5% had experienced episodes at least weekly during the last three months. Table 5 presents an overview of the lifetime prevalences of the six specific sexsomnias, showing that masturbation was the most common among the whole sample (5.4%), whereas consummated intercourse was the least common (1.8%). Although there was a male preponderance of sexsomnia, there were no significant sex differences in terms of the proportions who reported the six sexsomnias, among those who reported at least one sexsomnia. Both for men and women, masturbation was the most common sexual behavior, whereas consummated intercourse was the least common. Of those who reported lifetime sexsomnia, 23.2% confirmed none of the six specific sexsomnias listed, 29.1% confirmed one, 15.2% confirmed two, 15.6% confirmed three, 9.4% confirmed four, 6.2% confirmed five, and 1.4% confirmed all six.

**Table 1:** Overview of study variables (N = 1002)

Variable	Mean (SD) or percentage
Sex	
Males	50.1%
Females	49.9%
Age (years)	48.8 (17.9)
Sleep walking lifetime	18.5%
Sleep terrors lifetime	11.6%
Confusional arousals lifetime	19.3%
Dream enactment lifetime	20.1%
Sexsomnia lifetime^a^	10.5%
Sexsomnia (current; last 3 months)^a^	6.1%
Sleep debt per day	
No debt	36.3%
Less than 1 h debt	28.7%
1 h or more debt	35.0%

^a^34 participants did not want to answer these questions

**Table 2:** Logistic regression results for life-time sexsomnia (N = 969)

	Crude	Adjusted
Variable	OR (95% CI)	OR (95% CI)
Sex		
Females Male	1.00 **1.73 (1.14–2.61)**	1.00 **1.58 (1.03–2.42)**
Age (years)	1.00 (0.99–1.01)	1.01 (1.00–1.02)
Sleep walking lifetime	**1.79 (1.13–2.84)**	1.45 (0.88–2.40)
Sleep terrors lifetime	**3.87 (2.41–6.24)**	**2.80 (1.64–4.78)**
Confusional arousals lifetime	**2.12 (1.42–3.44)**	1.32 (0.79–2.21)
Dream enactment lifetime	**3.13 (2.04–4.80)**	**2.46 (1.56–3.88)**
Sleep debt per day No debt Less than 1 h debt 1 h or more debt	1.00 1.00 (0.60–1.66) 1.01 (0.63–1.63)	1.00 1.14 (0.70–1.94) 0.96 (0.58–1.60)

Note: Significant results are shown in bold

**Table 3:** Logistic regression results for current (last 3 months) sexsomnia (N = 969)

	Crude	Adjusted
Variable	OR (95% CI)	OR (95% CI)
Sex Females Male	1.00 1.46 (0.87–2.48)	1.00 1.36 (0.79–2.32)
Age (years)	1.00 (0.99–1.01)	1.00 (0.99–1.02)
Sleep walking lifetime	1.19 (0.63–2.26)	0.89 (0.45–1.76)
Sleep terrors lifetime	**3.74 (2.09–6.71)**	**2.86 (1.48–5.51)**
Confusional arousals lifetime	**2.09 (1.20–3.66)**	1.31 (0.69–2.50)
Dream enactment lifetime	**2.55 (1.48–4.41)**	**1.97 (1.10–3.51)**
Sleep debt per day No debt Less than 1 h debt 1 h or more debt	1.00 0.92 (0.47–1.82) 1.23 (0.67–2.24)	1.00 1.02 (0.51–2.05) 1.16 (0.62–2.15)

Note: Significant results are shown in bold

**Table 4:** Frequency of sexsomnia for the last 3 months among participants with lifetime sexsomnia (n = 105)

Never	42.1%
Less than once per month	41.7%
Less than once per week	9.8%
1–2 days per week	5.3%
3–5 days per week	1.2%
Daily or almost daily	0.0%

**Table 5:** Lifetime prevalence of various sexsomnias for the whole sample and for females and males with sexsomnia

Sexsomnnia	Whole sample	Women with sexsomnia	Men with sexsomnia	χ^2^ (df = 1)^1^
Masturbation	5.4% (n = 963)	51.3% (n = 39)	52.5% (n = 61)	0.00, n.s
Fondling	4.0% (n = 965)	35.0% (n = 40)	40.3% (n = 62)	0.10, n.s
Initiated intercourse	2.6% (n = 966)	23.1% (n = 39)	26.2% (n = 65)	0.01, n.s
Consummated intercourse	1.8% (n = 968)	17.5% (n = 40)	15.4% (n = 65)	0.00, n.s
Sexualized vocalization	2.1% (n = 962)	18.4% (n = 38)	21.3% (n = 61)	0.01, n.s
Sexualized behavior (e.g., pelvic thrusts)	3.7% (n = 962)	39.5% (n = 38)	32.8% (n = 61)	0.21, n.s

^1^Continuity correction, n.s. = not significant

## Discussion

The lifetime prevalence of sexsomnia in the present study was 10.5%, which is somewhat higher than reported by Bjorvatn et al. (2010), but somewhat lower than the 15.3% reported by Cankardas and Schenck (2021). The results suggest that a considerable amount of the general population reportedly experience sexsomnia. A considerable proportion, 6.5%, reported sexsomnia occurring during the last three months. Still, such self-reported surveys should be corroborated by clinical interview data, and it would also be of interest to link survey data to polysomnographic recordings. About 42% of the participants with lifetime sexsomnia did not report any episodes during the last three months. This might reflect cases of spontaneous recovery, successful treatment, or that periods with active symptoms may vary and be separated by periods without symptoms. So far not much is known about the natural trajectory of sexsomnia, hence longitudinal studies are called for. Males had a higher probability than females to report sexsomnia, which is in agreement with several previous studies (Andersen et al., 2024; Cankardas & Schenck, 2021; Chung et al., 2010). However, male sex was not related to current sexsomnia, maybe due to reduced statistical power. In the crude analyses, all the four other parasomnias (sleep walking, sleep terrors, confusional arousal, and dream enactment) were related to lifetime sexsomnia; however, only sleep terrors and dream enactment remained significant in the adjusted analyses. This epidemiological finding is mirrored by clinical findings of up to five additional comorbid parasomnias in patients with sexsomnia (Cicolin et al., 2011; Schenck, 2015; Schenck et al., 2007; Soca et al., 2016).

Except for a non-significant relationship with sleepwalking in the crude analysis, the other parasomnias were significantly associated with current sexsomnia, as well. Overall, this is in line with other studies showing a relationship with other NREM-related parasomnias and sexsomnia (Bjorvatn et al., 2010; Martynowicz et al., 2018), which might reflect some common underlying pathophysiology (Dubessy et al., 2017; Rossi et al., 2023). Interestingly, dream enactment was a consistent predictor of lifetime and current sexsomnia in the present study. This may in some cases reflect behavior from REM-sleep, and as such suggestive of REM-sleep behavior disorder or parasomnia overlap disorder (Cicolin et al., 2011; Soca et al., 2016). However, more recent evidence suggests that dream reports and objective dream enacting behaviors also occur during disorders of NREM arousal (Castelnovo et al., 2024; Idir et al., 2022), which may explain why dream enactment in the current and in previous studies (Cankardas & Schenck, 2021) has been associated with sexsomnia.

Despite the fact that sleep loss has long been identified as a risk factor for sexsomnia (Andersen et al., 2007; Riha et al., 2023), current sleep debt was neither a predictor of lifetime sexsomnia, nor of current sexsomnia. One reason for this is that the current survey assessed sleep debt generally, and did not take day to day variability of sleep duration into account.

In terms of frequency of current sexsomnia, the results indicated that for most of the participants with sexsomnia, these episodes occurred relatively rarely, as only 6.5% reported a weekly or more frequent occurrence. This frequency is lower than what has been reported in clinical samples referred to a sleep disorder center (Dubessy et al., 2017), which may suggest that frequency of sexsomnia may be a motivating factor for treatment seeking. The two most commonly reported sexsomnic behaviors in the present survey were masturbation and fondling, whereas consummated intercourse was the least common. Trajanovic et al. (2007) also found that masturbation and fondling were quite frequent in an internet survey among 219 adults (69% males). Still, those authors reported intercourse to be far more prevalent than in the present study, although they did not differentiate between initiating and consummating intercourse (Trajanovic et al., 2007). Among the participants with sexsomnia, we did not find any sex difference regarding the various sexsomnic behaviors; however, this might reflect low statistical power. Still, the sex differences were low for all the various sexsomnic behaviors in terms of the proportions, hence it seems reasonable to conclude that no meaningful sex differences appeared in the current sample. The vast majority (83.1%) of the participants with sexsomnia reported to have performed three or fewer of the six sexsomnic behaviors in question. This suggest that most of those with sexsomnia have a somewhat restricted behavioral repertoire during the episodes, although cases reflecting a full sexual repertoire during the same episode have been reported (Schenck et al., 2007). However, in this regard it should also be noted that we restricted the alternatives to six common sexsomnia variants, which do not fully cover the range of possible and maybe relevant sexual behaviors that might occur during sleep.

### Strengths and Limitations

The present study is one of few studies on the epidemiology of sexsomnia conducted in general population samples, and the sample size was large compared to most other comparable studies (Cankardas & Schenck, 2021; Trajanovic et al., 2007). Another asset of the present study was that we asked about lifetime as well as current sexsomnia, and also tapped into various sexsomnic behaviors. Still, some limitations deserve mention, such as the cross-sectional and retrospective design, as well as the sensitive topic, which may cause biases such as the common method bias (Podsakoff et al., 2003), recall bias (Raphael, 1987), and social desirability bias (Gnambs & Kaspar, 2015). Further, the accounts of sexsomnia were neither corroborated by clinical data nor partner reports. Another limitation was that no distinction between activities with and without orgasm/ejaculation was made in the present survey, hence this should be addressed by future studies. Due to the sensitive nature of the topic investigated, underreporting may be expected (King, 2022), although this bias is normally reduced when anonymity is ensured (Durant et al., 2002), as in the present study.

The sample was drawn from a survey panel and the response rate was low, hence the results cannot, without reservations, be generalized to the general population. About one fourth of those reporting sexsomnia did not endorse any of the specific sexsomnic behaviors listed, which may reflect that some sexual behaviors (like oral-genital sex, fingering/digital sex and anal sex) were not included, hence future epidemiological studies on sexsomnia should encompass a more comprehensive list of possible sexual acts.

### Conclusions

Sexsomnia seems to be more prevalent than previously assumed. A history of sleep terrors and dream enactment were consistent predictors of lifetime, as well as current, sexsomnia. Masturbation and fondling were the most common sleep related sexual behaviors, whereas consummated intercourse appeared as the least common. Among those reporting current sexsomnia, the frequency of occurrence was quite low, and most participants reported a restricted range of various sexual behaviors. Future studies, including clinical interviews, should be conducted with representative samples from the general population across multiple countries and geographic regions, and longitudinal studies enabling elucidation of temporal trajectories of sexsomnia are warranted. Studies should also aim to cover a wider range of possible sleep related sexual behaviors than those covered by previous studies and the current study, and more data on precipitating factors, time of occurrence during the day, marital and legal consequences, as well as treatment seeking behaviors should be included. Establishing consensus about how to assess the presence of sexsomnia in epidemiological contexts would enable more accurate comparisons of results across studies.

## Data Availability

The data are available by contacting the corresponding author.
